# Improving the Estimation of Celiac Disease Sibling Risk by Non-HLA Genes

**DOI:** 10.1371/journal.pone.0026920

**Published:** 2011-11-07

**Authors:** Valentina Izzo, Michele Pinelli, Nadia Tinto, Maria Valeria Esposito, Arturo Cola, Maria Pia Sperandeo, Francesca Tucci, Sergio Cocozza, Luigi Greco, Lucia Sacchetti

**Affiliations:** 1 Department of Pediatrics, University of Naples Federico II, Naples, Italy; 2 European Laboratory for Food Induced Disease, University of Naples “Federico II”, Naples, Italy; 3 Department of Cellular and Molecular Biology and Pathology “L. Califano”, University of Naples “Federico II”, Naples, Italy; 4 CEINGE Advanced Biotechnology, S.c.a.r.l., Naples, Italy; 5 Department of Biochemistry and Medical Biotechnology, University of Naples “Federico II”, Naples, Italy; University of North Carolina, United States of America

## Abstract

Celiac Disease (CD) is a polygenic trait, and HLA genes explain less than half of the genetic variation. Through large GWAs more than 40 associated non-HLA genes were identified, but they give a small contribution to the heritability of the disease. The aim of this study is to improve the estimate of the CD risk in siblings, by adding to HLA a small set of non-HLA genes. One-hundred fifty-seven Italian families with a confirmed CD case and at least one other sib and both parents were recruited. Among 249 sibs, 29 developed CD in a 6 year follow-up period. All individuals were typed for HLA and 10 SNPs in non-HLA genes: CCR1/CCR3 (rs6441961), IL12A/SCHIP1 and IL12A (rs17810546 and rs9811792), TAGAP (rs1738074), RGS1 (rs2816316), LPP (rs1464510), OLIG3 (rs2327832), REL (rs842647), IL2/IL21 (rs6822844), SH2B3 (rs3184504). Three associated SNPs (in LPP, REL, and RGS1 genes) were identified through the Transmission Disequilibrium Test and a Bayesian approach was used to assign a score (BS) to each detected HLA+SNPs genotype combination. We then classified CD sibs as at low or at high risk if their BS was respectively < or ≥ median BS value within each HLA risk group. A larger number (72%) of CD sibs showed a BS ≥ the median value and had a more than two fold higher OR than CD sibs with a BS value < the median (O.R = 2.53, p = 0.047). Our HLA+SNPs genotype classification, showed both a higher predictive negative value (95% vs 91%) and diagnostic sensitivity (79% vs 45%) than the HLA only. In conclusion, the estimate of the CD risk by HLA+SNPs approach, even if not applicable to prevention, could be a precious tool to improve the prediction of the disease in a cohort of first degree relatives, particularly in the low HLA risk groups.

## Introduction

Celiac disease (CD) is a chronic small-intestinal enteropathy, triggered by gluten proteins contained in wheat, barley and rye [1]. The evidence of a strong genetic component is suggested by a remarkable familiar aggregation: the prevalence of CD is, in fact, 10 times higher in first degree relatives (∼10%) than in the whole population (1%) [1]–[3] a and very high concordance (>80%) is found in monozygotic twins [4]. CD prevalence increased significantly in the last 20 years, so becoming a major public health problem, for this reason, in the near future the CD families are predicted to be a major source of new cases and consequently the CD risk prediction in these cohorts may be important.

At present Histocompatibility Leucocyte Antigens (HLA) explains ∼35% [5] of the genetic variance associated to CD. We previously graded the HLA risk genotype in 5 risk groups (from G1 to G5) and we were able to calculate the risk in each group with very wide confidence intervals (from 0.1% to ≥20%) [1]. In particular the higher risk groups (>10%) were those belonging to G1 and G2 groups [1]. However, since HLA alone can explain about 1/3rd of the genetic susceptibility to the disease, other variants should be implicated.

In the last four years, several Genome Wide Association studies (GWAs) identified about 40 genomic regions harboring 64 candidate genes, which are involved in adaptive and innate immunity in CD and also linked to other autoimmune diseases [6]–[13]. Unfortunately altogether non-HLA genes account for only 4% of the genetic variance [13].

In the field of complex diseases, particularly in CD, great attention is now paid to use the available genetic data to predict the risk of disease in asymptomatic individuals and to support the diagnosis in difficult cases. Although it was described that non-HLA genes improve the ability to identify individuals at high risk, the increased predict ability by only genetics seems still modest in the general population [14]. However the use of non-HLA genes in the disease risk prediction in CD sibs has not yet been explored.

The Bayesian approach was shown to be useful in the managing the GWAS results, for example, in predicting the susceptibility to breast cancer [15]. Applying the same approach in a CD family cohort, we wish to improve the estimation of CD risk among siblings of Coeliacs over the available risk HLA-based and thus provide a better tool to evaluate the health status or to predict the disease in these at risk individuals.

## Results

### SNPs evaluation and computation of the Bayesian Score

**Figure 1:**
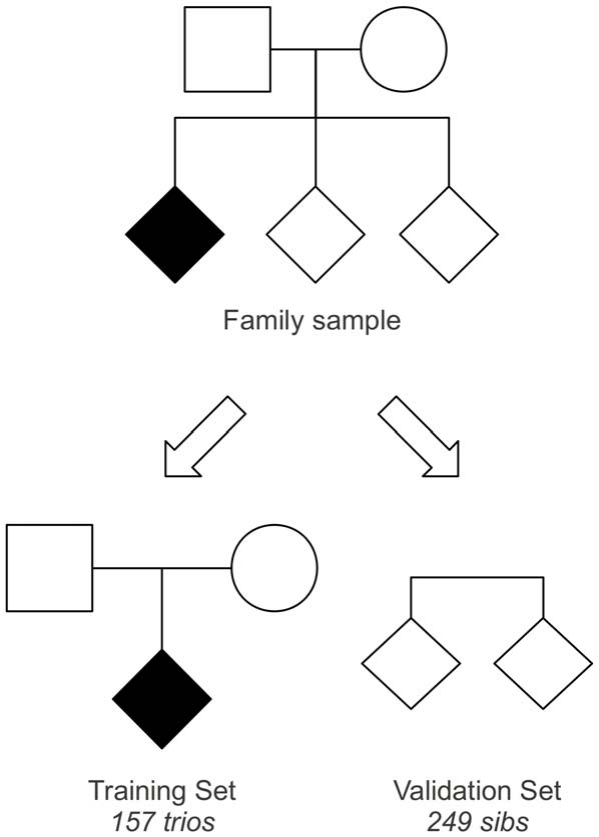
Study design of CD families. The family set was splitted in a Training set, that is 157 *trios* composed by the 157 probands and their unaffected parents and in a Validation set, that is 249 sibs of the probands.

All individuals were typed for 10 CD previously associated SNPs [13] and the TDT test was performed on results obtained from 157 trios of the training set (Fig. 1). Three out of ten investigated SNPs (rs1464510 in LPP, rs842647 in REL and rs2816316 in RGS1 genes) were significantly associated with CD (Table 1). In particular: rs1464510 in LPP gene showed a strong association (p<0.001) according to an additive model, whereas, both rs2816316 in RGS1 and rs842647 in REL genes were also significantly associated (respectively p = 0.025 and p = 0.034), by a recessive model. For supporting information about allelic and genotypic frequencies observed in the sample see Table S1 and S2.

**Table 1:** Genotypic Transmission Disequilibrium Test (TDT) results.

SNP	Gene	Model	Risk Allele	OR (95% CI)	p value
rs1464510	LPP	Additive	A	2.36 (1.64–3.41)	<0.001
rs2816316	RGS1	Recessive	A	1.75 (1.07–2.86)	0.025
rs842647	REL	Recessive	A	1.66 (1.04–2.65)	0.034
rs2327832	OLIG3	Additive	G	1.35 (0.90–2.03)	0.150
rs6441961	CCR1/CCR3	Additive	A	1.24 (0.89–1.72)	0.189
rs6822844	IL2/IL21	Additive	C	1.43 (0.82–2.49)	0.210
rs1738074	TAGAP	Dominant	A	1.31 (0.79–2.16)	0.293
rs3184504	SH2B3	Additive	A	1.19 (0.86–1.63)	0.294
rs17810546	IL12A	Additive	G	1.10 (0.80–1.51)	0.572
rs9811792	IL12A/SCHIP1	Dominant	G	1.10 (0.59–2.05)	0.753

For supporting information about allelic frequencies observed in Trios (Probands and unaffected parents) and in sibs (affected and unaffected) see Table S1.

In order to evaluate the occurrence of HLA-SNP interaction, we stratified the training set (Fig. 1) according to the HLA risk group of the proband. No statistically significant interaction was found between HLA and non-HLA genes (data not shown).

To compute the Bayesian Score (BS) we compared the frequency of each HLA+SNPs genotype combination detected in probands and in controls (Training set, Fig. 1). Through this approach we obtained a BS for each HLA+SNPs genotype combination (data not shown).

### Validation of the BS and testing of a classification model

The validation set (Fig. 1) was composed by the sibs of the probands, both affected (n = 29) and unaffected (n = 220). In these subjects we evaluated if HLA+SNPs genotyping could improve the identification of CD risk in sibs better than HLA only.

**Figure 2:**
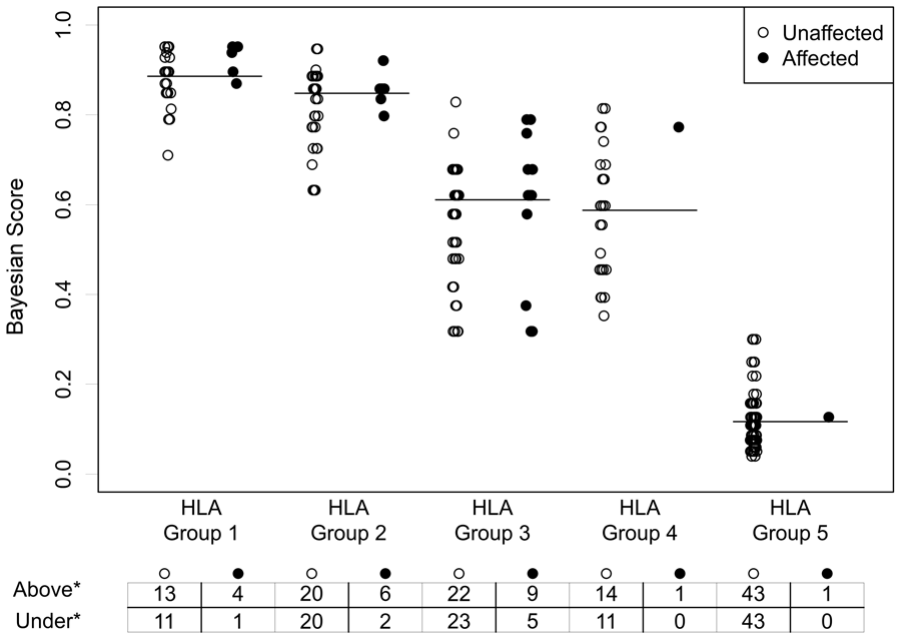
Distribution of CD sibs based on their BS. Affected (n = 29) and unaffected (n = 220) CD sibs were classified on the basis of their BS value < or ≥ median BS within each HLA group. Horizontal lines correspond to the median BS of all sibs in each HLA risk-group (HLA Group 1 = 0.90, HLA Group 2 = 0.86, HLA Group 3 = 0.62, HLA Group 4 = 0.60, HLA Group 5 = 0.13).

We assigned to each sib, both affected and unaffected, the BS value corresponding to his HLA+SNPs genotype combination as previously calculated in the training set (Table 2). We observed an increase of average BS values from HLA group 5 to HLA group 1 and, within all 5 HLA groups we found an increase of the BS corresponding to an increase of “A” alleles in the haplotype combination. In order to identify sibs at high risk to develop CD, we distributed affected and unaffected sibs on the basis of their BS (under or above median BS) within each HLA group from G1 to G5 (Fig. 2). Considering the distribution of all the sibs, it is evident that above the median BS within each HLA group, there was always a larger number of affected sibs than under the median: 72% (21/29) versus 28% (8/29). Interestingly, 2/29 affected sibs that being at lower HLA risk (HLA group 4 and 5) could be misclassified, were correctly classified by their BS above the median. We calculated the Odds Ratio (OR) of CD classification based on the BS, above or under the median, within each HLA group. CD sibs with a BS value above the median, had more than two fold higher risk (OR) compared to CD sibs with a BS value under the median (2.53, 95% C.I.: 1.68–3.39; p = 0.047).

**Table 2:** Bayesian Score (BS) assigned to each HLA-SNPs genotype combination.

Associated SNPs			BS				
LPP(rs1464510)	REL(rs842647)	RGS1(rs2816316)	HLA Group 1	HLA Group 2	HLA Group 3	HLA Group 4	HLA Group 5
CC	AG|GG	AC|CC	0.66	0.63	0.32	0.30	0.04
CC	AG|GG	AA	0.74	0.73	0.42	0.39	0.06
CC	AA	AC|CC	0.71	0.69	0.38	0.35	0.05
CC	AA	AA	0.79	0.77	0.48	0.46	0.08
AC	AG|GG	AC|CC	0.81	0.80	0.52	0.49	0.09
AC	AG|GG	AA	0.87	0.86	0.62	0.60	0.13
AC	AA	AC|CC	0.85	0.84	0.58	0.55	0.11
AC	AA	AA	0.90	0.89	0.68	0.66	0.16
AA	AG|GG	AC|CC	0.91	0.90	*	0.69	0.18
AA	AG|GG	AA	0.94	0.93	0.79	0.77	0.25
AA	AA	AC|CC	0.93	0.92	0.76	0.74	0.22
AA	AA	AA	0.95	0.95	0.83	0.81	0.30

*The combination LPP*AA - REL*AG|GG - RGS1*AC|CC for HLA group 3 was not found among our sibs' cohort.

**Figure 3:**
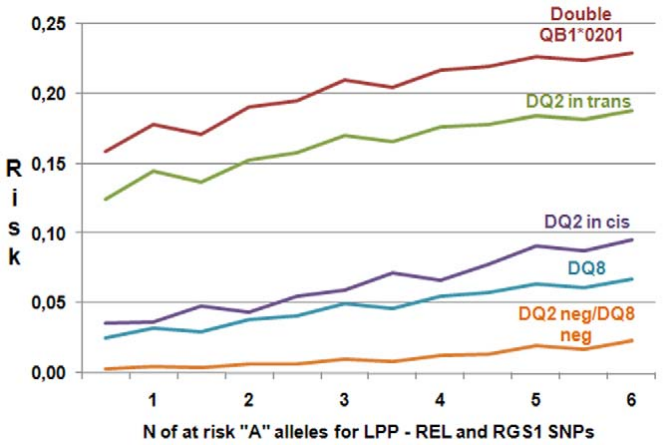
Refining the CD risk estimate. The picture shows the modification of the *a priori* HLA related risk by the number of the at risk “A” alleles of the LPP, REL and RGS1 SNPs. From top to bottom lines correspond to HLA group 1 to 5.

In our previous work, we estimated the HLA related risk to develop CD, defining a risk range from 0.01 to 0.21 [1]. To refine the previous HLA based estimation of risk by this new approach we set the HLA related mean risk as the *a priori* risk of the Bayesian Model. Having set the HLA risk at the median level of the Bayesian Score, we produced a picture of the variation of risk given by the non-HLA genes for each HLA class (Fig. 3). It is remarkable that the addition of the 3 SNPs does modify the HLA only risk through the 5 HLA risk classes, also in subjects with no DQ2 neither DQ8 haplotype, where the increase in risk is significantly larger than that observed in HLA risk classes.

The addition of only 3 SNPs to the HLA significantly improved the prediction of CD risk in sibs, identifying, within a specific HLA group, those individuals which are more likely to become celiacs. In fact, considering HLA 1 and 2 as the highest risk groups [1], we calculated the diagnostic characteristics of our proposed HLA+SNPs genotype combination obtaining both an higher predictive negative value (NPV) and an higher diagnostic sensitivity (DS) than HLA only, respectively 95% vs 91% (NPV) and 79% vs 45% (DS) (Table 3). Although the discovery of 39 polymorphism associated to CD improved the estimation of the heritability of only 4–5% [13], in this Bayesian model the addition of only three SNPs of associated genes improves the sensitivity of risk prediction of 34% compared to the HLA only model.

**Table 3:** Diagnostic characteristics of HLA and HLA-SNPs genotype combination.

	HLA risk groups 1–2	HLA-SNPs genotype combination
**Sensitivity**	0.45 (0.27–0.64)	0.79 (0.73–0.84)
**Specificity**	0.71 (0.65–0.77)	0.54 (0.48–0.60)
**NPV**	0.91 (0.87–0.96)	0.95 (0.92–0.98)
**PPV**	0.17 (0.12–0.22)	0.19 (0.14–0.24)

The computation of the AUC of the ROC curve output a C statistic equal to 0.70 for HLA and 0.73 for HLA+SNPs classifications, showing that the inclusion of SNPs moderately improved the prediction ability.

## Discussion

In our previous work, we considered in CD families the risk to develop the disease according to a specific HLA haplotypes, obtaining a risk range from 0.01 to ≥0.20 [1]. In the present study we evaluated the role of 3 non-HLA genetic markers to influence the CD risk in first relatives of CD affected children.

We collected data on families with at least one CD-affected among offspring. This family set helped to evaluate the association between SNPs and CD (TDT on parents-offspring trios) and to estimate the risk of CD in the other sibs. The TDT design provides robustness to population stratification and mitigation of the possible confounding effect of environmental factors, because all family members share the same environment [16].

Ten SNPs, selected from those previously found to be associated with CD by GWAS [13], were successfully genotyped. In our population three SNPs resulted significantly associated with CD (those in LPP, RGS1 and REL genes) and the other seven investigated SNPs, even if not statistically associated with CD, showed always an higher frequency of the previously reported risk alleles [13] in affected subjects than in controls.

The three genes selected appear to be appealing for the pathogenesis of CD. LPP (OR = 2.36; p<0.001) was reported to be highly expressed in small intestinal mucosa and may have a structural role at sites of cell adhesion in maintaining cell shape and motility [7]. RGS1 (OR = 1.75; p = 0.025) belongs to a family of RGS genes. It attenuates the signaling activity of G-proteins, blocking the homing of Intra Epithelial Lymphocytes (IELs), and it is specifically expressed both in human small intestinal mucosa and in murine IELs, key players in the development of human CD villous atrophy [7], [17]. REL (OR = 1.66; p = 0.034) is a subunit of NF-kB complex, implicated in T cell differentiation [18] and it appears to be a key molecule regulating inflammation and the switch from tolerance to autoimmunity [19]. It is interesting to note that our data confirm previous pathogenetic implications reported in literature of these SNPs with CD as well as with other autoimmune diseases [20].

By the Bayesian approach we calculated a ranking score (BS) among the sibs. However, it should be considered that BS is not a plain disease risk, rather a method to rank different genotypes according to their contribution to make an individual susceptible to CD. For instance, some of our BS are very near to 1, nevertheless none of the considered genotypes could give a 100% risk to develop the disease. In other terms, we considered the BS as a ranking measure, only stating that a given genotype could assign a higher risk than another genotype but does not allow a quantitative measure of the risk difference (2-fold, 3-fold, etc). However, even if the addition of only 3 SNPs to HLA could be considered at “minor effect” [13], we demonstrated that they could significantly improve the prediction of CD risk in sibs, in terms of diagnostic sensitivity and negative predictive value. So, in a cohort of CD families, our data confirm that non-HLA SNPs evaluation is an usefull diagnostic tool in CD risk evaluation as a previous study showed in CD unrelated subjects [14].

CD, on the basis of the actual knowledge, cannot be exactly predicted by genetic testing, but a reliable probabilistic method might be associated to careful surveillance of infants carrying the higher risk. This will help to significantly reduce the heavy load of anxiety and pain associated with the appearance of symptoms of CD, by anticipating, with simple serological tests, the clinical appearance of the disease.

**Figure 4:**
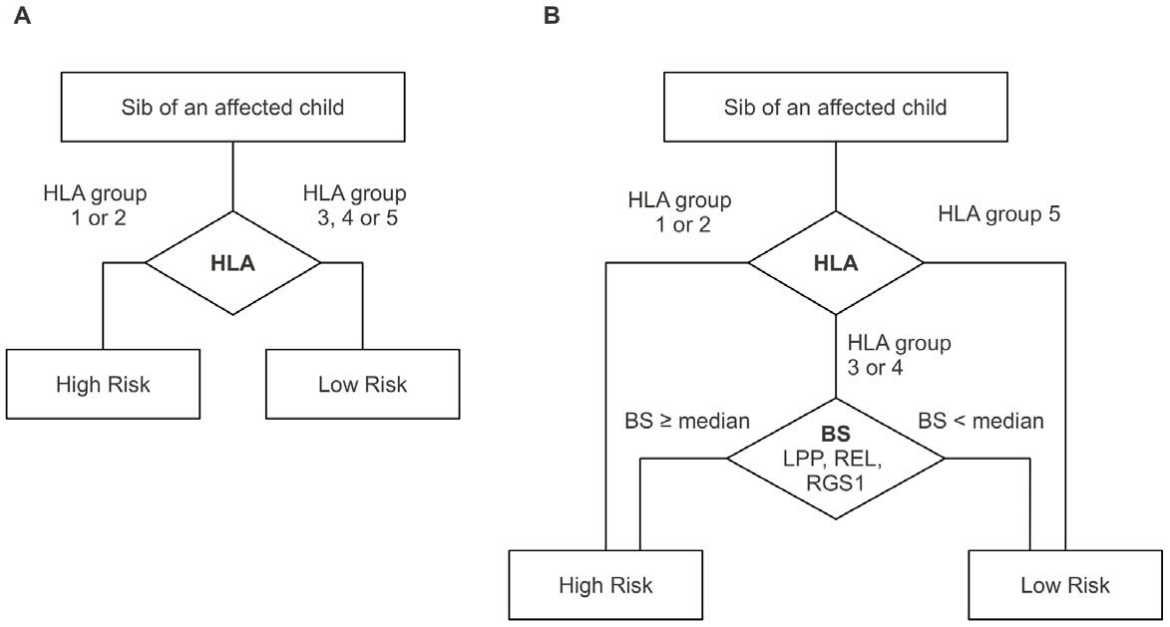
Classification flow-chart. In panel A the classical HLA-based classification. In panel B the proposed BS-based classification considering the genotypes of HLA plus LPP, RGS1 and REL SNPs.

To improve the possibility to identify high risk patients in CD families we propose in alternative to the classical HLA classification (Fig. 4, panel A) a slight improved flow-chart (Fig. 4, panel B): 1) HLA genotyping: subjects belonging to the HLA risk groups 1 and 2 will be classified as at high CD risk; 2) subjects belonging to the HLA risk groups 3 and 4, will be further investigated for our SNPs combination (LPP, REL, RGS1) in order to calculate their BS (Fig. 4, panel B). Among these latter subjects those with a BS ≥ the median value will be classified at high risk; 3) subjects belonging to the HLA risk group 5 will be considered at low CD risk. All CD familials belonging to the above high risk groups (HLA group 1–2 and HLA group 3–4 with BS ≥ median) will be undergo a strict surveillance.

One of the limitation of our cohort family study could be the sample size, which may have not allowed to explore genes at smaller effect, so explaining the lack of association between SNPs in TAGAP, IL2/IL21, OLIG3, CCR, SH2B3, IL12A and IL12A/SCHIP1 genes with CD although the trend observed in previous studies in unrelated CD patients was confirmed [13]. In the main time the homogeneity of the genetic and environmental domains in the tested families allows to explore risk factors within a controlled cohort. A second limit of the study is the relatively short (6 years) follow up of the sibship, which could cause an underestimation of the disease development at later ages. Our aim is to go on with the monitoring of these families in the next years.

In conclusion, the estimate of the CD risk by HLA+SNPs approach, even if not applicable to prevention, could be a precious tool improving CD diagnosis respect to the only HLA (NPV: 95% vs 91%, and DS: 79% vs 45%), in the cohort of first degree relatives. In fact in clinical practice the absence of HLA risk groups 1 or 2, allows to exclude the disease with high probability, while testing the three SNPs in HLA groups 3 or 4 could represent a further tool to identify less frequent CD cases. So, an infant with high HLA+SNPs score even if belonging to HLA low risk groups, shall undergo a simple surveillance system to allow proper diagnosis and treatment before the full blow disease appears.

## Materials and Methods

### Ethic Statement

The written informed consent was obtained from all participants and from both parents for children. The research was approved by the Ethics Committee of the School of Medicine, University of Naples “Federico II” and was according to principles of the Helsinki II declaration.

### Family Samples

A cohort of CD families was recruited as previously described [1]. Families included a symptomatic CD patient (hereafter referred as the proband), both parents and at least one sib (for a total of 183 probands, 366 parents and 249 sibs); all probands, as well as the new cases, were diagnosed according to the European Society of Paediatrics Gastroenterology and Nutrition (ESPGHAN) criteria [21]. Among the 249 sibs, 29 resulted to be affected over a 6 years follow up program [1].

All individuals were grouped into five decreasing risk classes according to their HLA genotype: very high (>20% with two copies of DQ2.5, or DQ2.5/DQ2.2 Group 1), high (15–20% with DQ2.2/DQA105, Group 2), intermediate (10–15% with one copy of the DQ2.5 heterodimer, Group 3), moderate (1–10% with a double copy of DQ8 or DQ2.2/DQ8, or double copy of DQ2.2, Group 4) or negligible (<1%, with other genotype, Group 5) (Table S3) [1].

### Non-HLA Single Nucleotide Polymorphisms (SNP) typing

The 798 patients were genotyped for 10 non-HLA SNPs associated with CD: rs6441961 on 3p21 (*Chemokine C-C motif receptor 1 and 3* – CCR1/CCR3), rs17810546 and rs9811792 on 3q25-26 (SCHIP1 – *Schwannomin interacting protein 1* – and IL12A – *Interleukin 12A*), rs1738074 on 6q25 (*T cell activation GTPase activating protein* – TAGAP), rs2816316 on 1q31 (*Regulator of G-protein signaling 1* – RGS1), rs1464510 on 3q28 (*Lipoma Preferred Partner* – LPP), rs2327832 on 6q23.3 (*Oligodendrocyte transcription factor 3* – OLIG3), rs842647 on 2p16.1 (*Reticuloendotheliosis viral oncogene homolog* – REL) , rs6822844 on 4q27 (*Interleukin 2 and 21* – IL2/IL21), rs3184504 on 12q24 (*SH2B adaptor protein 3* – SH2B3). Genotyping reactions were performed using TaqMan®SNP Genotyping Assays on a 7900HT Fast Real-Time PCR System (Applied Biosystems, Foster City, CA, USA); the final volume was 5 µL, containing master mix, TaqMan assays and about 60 ng of genomic DNA template. All 384 well plates were filled using Biomek® FX (Beckman Coulter, Indianapolis, IN, USA). Allelic Discrimination results were analyzed through the SDS software ver. 2.3.

### Analysis strategy and statistics

In order to develop the model we splitted the sample into a training and a validation set (Fig. 1). In the *training set*, composed by probands and their unaffected parents, called *trios*, we evaluated which SNP was associated to the disease, independently from the HLA haplotype. Twenty-six CD families were excluded because they did not meet the requirements for the analysis (e.g. there was an affected parent or patients had an incomplete genotyping), so the training set was composed by 157 trios (Fig. 1). As control haplotypes we considered the haplotypes carried by parents and not transmitted to the affected probands, as they could be representative of the haplotypes in the general population, from which cases are originated. The frequencies of HLA and non HLA haplotypes in controls were estimated by the AFBAC (Affected Family Based Controls) method implemented in the MASC software tool [22]. To evaluate the association of the SNPs with the disease, we performed a *Transmission Disequilibrium Test* (TDT) [16] by using the *trio* package ver. 1.1.15 for R statistical computing software ver. 2.11.2. This method is based on multivariate logistic regression. We considered: dominant, the heterozygote genotype when it had the same risk effect of the high risk homozygote genotype; recessive, the heterozygote genotype when it had the same risk effect of the low risk homozygote genotype; additive, the heterozygote genotype when it had a risk effect intermediate between the two homozygote genotypes. Firstly, we evaluated monovariate association between each SNP and the disease. Secondly, among those resulted to be significantly associated with CD, we evaluated for SNP-SNP interactions by logistic regression model. Finally, the interaction between significant SNPs and HLA groups was verified. In details, we repeated association statistics between CD risk and SNPs on different strata of HLA groups and, thus, computed the interaction tests.

To evaluate the join effect of HLA and SNPs to influence the risk to develop CD in sibs, we implied a Bayesian approach, focusing only on those SNPs resulted significantly associated with CD at TDT analysis. Usually Bayes' revision of probabilities allows the computation of an individual probability of an event given the *a priori* data from the general population where the patient comes from. We arbitrarily selected an *a priori* probability of 0.5, because in our situation we could difficulty apply the Bayes' revision of probabilities and obtain an a posteriori risk of CD in sibs. Indeed the estimated 10% of CD risk in first grade relatives also account for the role of the genetics, therefore it cannot be used as an a priori probability to calculate the a posteriori probability, after considering the risk conferred by genetic factors (SNPs).

Firstly we considered the different frequencies of HLA genotypes in probands and controls and secondly the non-HLA SNPs genotypes frequencies. According to the Bayesian approach, in each run we used the score obtained in the previous step as the new *a priori* value for the following step (see Text S1). By this approach, it was possible to assign to each combination of HLA plus LPP, RGS1 and REL SNPs genotypes a BS. Secondly, we assigned to each sib (validation set) a BS in dependence of the specific HLA+SNPs genotype combination found. We arbitrarily established the median BS as discrimination threshold between low and high risk sibs and evaluated how affected and unaffected sibs were distributed. We performed C statistic by using the R “ROCR” statistic package to strength the interpretation of the results.

In order to produce a more realistic Bayesian Risk Score, we then considered the mean HLA related risk [1] as the a priori risk (instead of 0.5) to be fit in the first step of the Bayesian equation. The corresponding scores (higher or lower than the median) were standardized by the starting HLA related risk.

## Supporting Information

Table S1 Allelic frequencies observed in Training set (Trios) and in Validation set (sibs).
(DOC)

Table S2 Non-HLA genotypic frequencies and HLA groups frequencies observed in Training set (Trios) and in Validation set (sibs).
(DOC)

Table S3 Classification according to the HLA genotype [1].
(DOC)

Text S1 Bayesian theorem.
(DOC)
