# Supplementary material for: Improving the Estimation of Celiac Disease Sibling Risk by Non-HLA Genes
Source: PLoS One. 2011 Nov 7;6(11):e26920. doi: 10.1371/journal.pone.0026920 (PMC3210127; doi:10.1371/journal.pone.0026920)
Supplement: Table S1 — Allelic frequencies observed in Training set (Trios) and in Validation set (sibs). (DOC) [file pone.0026920.s001.doc]

**Table S1**

|  |  | **Training set** | | **Validation set** | |
| --- | --- | --- | --- | --- | --- |
|  |  | **Probands** | **Parents** | **Affected** | **Unaffected** |
| **LPP** | **A** | 0.52 | 0.43 | 0.59 | 0.42 |
|  | **C** | 0.48 | 0.57 | 0.41 | 0.58 |
| **OLIG3** | **A** | 0.76 | 0.79 | 0.81 | 0.85 |
|  | **G** | 0.24 | 0.21 | 0.19 | 0.15 |
| **RGS1** | **A** | 0.88 | 0.85 | 0.84 | 0.83 |
|  | **C** | 0.12 | 0.15 | 0.16 | 0.17 |
| **REL** | **A** | 0.77 | 0.75 | 0.69 | 0.80 |
|  | **G** | 0.23 | 0.25 | 0.31 | 0.20 |
| **SH2B3** | **A** | 0.55 | 0.53 | 0.66 | 0.53 |
|  | **G** | 0.45 | 0.47 | 0.34 | 0.47 |
| **CCR** | **A** | 0.41 | 0.39 | 0.38 | 0.34 |
|  | **G** | 0.59 | 0.61 | 0.62 | 0.66 |
| **IL2/IL21** | **A** | 0.09 | 0.11 | 0.50 | 0.10 |
|  | **C** | 0.91 | 0.89 | 0.50 | 0.90 |
| **IL12A** | **A** | 0.57 | 0.58 | 0.64 | 0.56 |
|  | **G** | 0.43 | 0.42 | 0.36 | 0.44 |
| **TAGAP** | **A** | 0.42 | 0.41 | 0.40 | 0.39 |
|  | **G** | 0.58 | 0.59 | 0.60 | 0.61 |
| **SCHIP1** | **A** | 0.92 | 0.93 | 0.95 | 0.92 |
|  | **G** | 0.08 | 0.07 | 0.05 | 0.08 |
