# Supplementary material for: Improving the Estimation of Celiac Disease Sibling Risk by Non-HLA Genes
Source: PLoS One. 2011 Nov 7;6(11):e26920. doi: 10.1371/journal.pone.0026920 (PMC3210127; doi:10.1371/journal.pone.0026920)
Supplement: Table S2 — Non-HLA genotypic frequencies and HLA groups frequencies observed in Training set (Trios) and in Validation set (sibs). (DOC) [file pone.0026920.s002.doc]

**Table S2**

|  |  | **Training set** | | **Validation set** | |
| --- | --- | --- | --- | --- | --- |
|  |  | **Probands** | **Parents** | **Affected** | **Unaffected** |
| **LPP** | **AA** | 0.26 | 0.20 | 0.31 | 0.15 |
|  | **AC** | 0.51 | 0.45 | 0.55 | 0.55 |
|  | **CC** | 0.23 | 0.35 | 0.14 | 0.31 |
| **OLIG3** | **AA** | 0.57 | 0.64 | 0.62 | 0.70 |
|  | **AG** | 0.37 | 0.30 | 0.38 | 0.30 |
|  | **GG** | 0.06 | 0.06 | 0.00 | 0.00 |
| **RGS1** | **AA** | 0.77 | 0.71 | 0.69 | 0.66 |
|  | **AC** | 0.21 | 0.27 | 0.31 | 0.34 |
|  | **CC** | 0.02 | 0.02 | 0.00 | 0.00 |
| **REL** | **AA** | 0.62 | 0.58 | 0.38 | 0.59 |
|  | **AG** | 0.31 | 0.34 | 0.62 | 0.41 |
|  | **GG** | 0.07 | 0.08 | 0.00 | 0.00 |
| **SH2B3** | **AA** | 0.29 | 0.29 | 0.38 | 0.25 |
|  | **AG** | 0.52 | 0.50 | 0.55 | 0.57 |
|  | **GG** | 0.19 | 0.22 | 0.07 | 0.18 |
| **CCR** | **AA** | 0.17 | 0.15 | 0.10 | 0.11 |
|  | **AG** | 0.47 | 0.47 | 0.55 | 0.46 |
|  | **GG** | 0.36 | 0.38 | 0.34 | 0.43 |
| **IL2/IL21** | **AA** | 0.00 | 0.03 | 0.45 | 0.00 |
|  | **AC** | 0.18 | 0.17 | 0.09 | 0.19 |
|  | **CC** | 0.82 | 0.80 | 0.45 | 0.81 |
| **IL12A** | **AA** | 0.33 | 0.33 | 0.38 | 0.27 |
|  | **AG** | 0.47 | 0.49 | 0.52 | 0.58 |
|  | **GG** | 0.20 | 0.18 | 0.10 | 0.15 |
| **TAGAP** | **AA** | 0.18 | 0.15 | 0.14 | 0.11 |
|  | **AG** | 0.46 | 0.53 | 0.52 | 0.56 |
|  | **GG** | 0.35 | 0.32 | 0.34 | 0.33 |
| **SCHIP1** | **AA** | 0.84 | 0.86 | 0.90 | 0.85 |
|  | **AG** | 0.16 | 0.14 | 0.10 | 0.15 |
|  | **GG** | 0.00 | 0.01 | 0.00 | 0.00 |
| **HLA** | **Group 1** | 0.22 | 0.09 | 0.17 | 0.11 |
|  | **Group 2** | 0.42 | 0.12 | 0.31 | 0.18 |
|  | **Group 3** | 0.23 | 0.25 | 0.45 | 0.21 |
|  | **Group 4** | 0.08 | 0.11 | 0.03 | 0.11 |
|  | **Group 5** | 0.04 | 0.42 | 0.03 | 0.39 |
