# Supplementary material for: Improving the Estimation of Celiac Disease Sibling Risk by Non-HLA Genes
Source: PLoS One. 2011 Nov 7;6(11):e26920. doi: 10.1371/journal.pone.0026920 (PMC3210127; doi:10.1371/journal.pone.0026920)
Supplement: Table S3 — Classification according to the HLA genotype [1]. (DOC) [file pone.0026920.s003.doc]

**Table S3**

| **HLA risk Group** | **DR in linkage** | **DQ** |
| --- | --- | --- |
| **Group 1** | DR3/DR3  DR3/DR7 | DQ2.5/DQ2.5  DQ2.5/DQ2.2 |
| **Group 2** | DR5/DR7 | DQ7/DQ2.2 |
| **Group 3** | DR3/DR5  DR3/DR4  DR3/DRX* | DQ2.5/DQ7  DQ2.5/DQ8  DQ2.5/DQX |
| **Group 4** | DR7/DR7  DR7/DR4  DR4/DR4 | DQ2.2/DQ2.2  DQ2.2/DQ8  DQ8/DQ8 |
| **Group 5** | Others |  |
| X* different from 3, 4, 5, 7  DQ2.5 → DQA1*05 – DQB1*0201  DQ2.2 → DQA1*02 – DQB1*0202  DQ7 → DQA1*05 – DQB1*03  DQ8 → DQA1*0301 – DQB1*0302 | | |
