# Supplementary material for: Improving the Estimation of Celiac Disease Sibling Risk by Non-HLA Genes
Source: PLoS One. 2011 Nov 7;6(11):e26920. doi: 10.1371/journal.pone.0026920 (PMC3210127; doi:10.1371/journal.pone.0026920)
Supplement: Text S1 — Bayesian theorem. (DOC) [file pone.0026920.s004.doc]

**Text S1**

*Bayesian theorem*

P(A|B)=[P(B|A)*P(A)] / {P(B|A)*P(A)+P(B|Ā)*P(Ā)}

which is the rate of the frequency of the risk factor in the cases versus the frequency of the same factor in the controls.

We use the following notations:

A = Case

Ā = Pseudo-control

B = Risk factor
